# Supplementary material for: StratoMod: predicting sequencing and variant calling errors with interpretable machine learning
Source: Commun Biol. 2024 Oct 13;7:1316. doi: 10.1038/s42003-024-06981-1 (PMC11471861; doi:10.1038/s42003-024-06981-1)
Supplement: Supplementary file 2 — Supplementary Information [file 42003_2024_6981_MOESM2_ESM.pdf]

Supplemental Notes

Supplemental Note 1

The following was used to generate the gene counts for **Supplemental File 1.**

```
intersectBed -c -a GRCh38_mrg_full_gene.bed -b patho_hifi_ilm.tsv > \
    GRCh38_mrg_full_gene_FNcounts.bed

grep Illumina patho_hifi_ilm.tsv | \
    intersectBed -c -a GRCh38_mrg_full_gene.bed -b stdin > \
    GRCh38_mrg_full_gene_FNcountsIllonly.bed

grep both patho_hifi_ilm.tsv | \
    intersectBed -c -a GRCh38_mrg_full_gene.bed -b stdin > \
    GRCh38_mrg_full_gene_FNcountsboth.bed

grep SNV patho_hifi_ilm.tsv > patho_hifi_ilm_SNV.tsv

intersectBed -c -a GRCh38_mrg_full_gene.bed -b patho_hifi_ilm_SNV.tsv > \
    GRCh38_mrg_full_gene_SNV_FNcounts.bed

grep Illumina patho_hifi_ilm_SNV.tsv | \
    intersectBed -c -a GRCh38_mrg_full_gene.bed -b stdin \
    > GRCh38_mrg_full_gene_SNV_FNcountsIllonly.bed

grep both patho_hifi_ilm_SNV.tsv | \
    intersectBed -c -a GRCh38_mrg_full_gene.bed -b stdin > \
    GRCh38_mrg_full_gene_SNV_FNcountsboth.bed

paste GRCh38_mrg_full_gene_FNcounts.bed \
    GRCh38_mrg_full_gene_FNcountsIllonly.bed \
    GRCh38_mrg_full_gene_FNcountsboth.bed \
    GRCh38_mrg_full_gene_SNV_FNcounts.bed \
    GRCh38_mrg_full_gene_SNV_FNcountsIllonly.bed \
    GRCh38_mrg_full_gene_SNV_FNcountsboth.bed \
    GRCh38_mrg_full_gene_merged_FNcounts.txt
```

Where `GRCh38_mrg_full_gene.bed` is a list of all genes in GRCh38 (found in https://github.com/usnistgov/giab-cmrg-benchmarkset/blob/master/data/manually_created_files/GRCh38_mrg_full_gene.bed) and `patho_hifi_ilm.tsv` (**Supplemental File 2**) is a list of pathogenic variants with >90% likelihood of being missed by Hifi or Illumina as calculated by Stratomod.

Supplemental Note 2

Predicting Precision in PCR-free vs PCR-plus

We asked if StratoMod could be used to predict where Illumina PCR-plus and PCR-free sequencing technologies have higher sequencing or mapping error rates that could produce incorrect variant calls (ie lower precision) (**Supp Fig 6a**). This choice in comparison was motivated by the fact that PCR amplification is known to produce insertions and deletions in homopolymer repeats (stutter[1]) (**Supp Fig 6b**) and thus we hypothesized that the model would be able to precisely show the effect of homopolymer length on error rate, in addition to other repetitive genomic contexts.

We trained two models (for SNVs and INDELs) using Illumina PCR-free/plus VCF files. These models differed from those previously trained in “use-case 1” above in several ways. First, we used FP instead of FN as the error class, since in this case we were concerned with sequencing error modalities that could falsely give rise to variants. Second, we used all candidate sites (before filtering) from the DeepVariant VCFs since DeepVariant would likely filter out the sequencing errors we wished to interrogate. Third, we included DP and VAF as features in our model, bringing the total feature count up to 24 (see **Supp Table 1**). For these models, we observed that both precision and recall (measured by AUC) were similar between HG004 and HG007 (with HG007 lagging slightly behind as expected given it was the holdout dataset) (**Supp Fig 6c, Supp Fig 7**). The negative class in the training sets for SNV and INDEL were 63% and 85% percent respectively (**Supp Table 2**).

Overall, the largest driving features (unsurprisingly) were VAF and DP as read from the input VCF files (**Supp Fig 7**), as many errors had low VAF and abnormally low or high DP (**Supp Fig 8**). However, other features with large effect included homopolymer length and homopolymer imperfect fraction (**Supp Fig 7a**). When observing the homopolymer length feature profiles directly from the model, we found that the precision generally increased with increasing length as expected from PCR stutter and sequencing biases. For INDELs, PCR-plus generally predicted more errors, with relatively small interactions between PCR and homopolymer length (**Supp Fig 6d**). Most homopolymers fell between the lengths of 0 to 50 or 0 to 15 bp for A/T and G/C homopolymers respectively, with the number of variants decreasing exponentially with increasing length and ~100x more variants in A/T than in G/C homopolymers longer than 10 bp (**Supp Fig 9**). Previous work had found that the number of incorrectly called INDELs in A/T homopolymers was much larger than in G/C homopolymers,[2] which was reflected in the feature rankings by Stratomod, but Stratomod also showed that G/C homopolymer length similarly predicts more incorrectly called variants. Additionally, these feature plots provide more precise information regarding the length at which a certain relative error threshold will be crossed. In the case of SNVs, A homopolymers became more error-prone compared to the non-homopolymer baseline (the dotted lines in **Supp Fig 6d**) after 10 bp; G homopolymers of any length were more error-prone (note that both A and G homopolymer profiles were similar to their complements, see **Supp Fig 9**). Increased SNV error rates in G/C homopolymers have been attributed to inhibition of base elongation in GC-rich regions during sequencing by synthesis[3] or to formation of non-B-DNA stem-loop motifs at G quadruplexes.[4] For INDELs, these thresholds were conditional on the sequencing technology, where PCR-free and PCR-plus were more error-prone than baseline after 13 and 11 bp respectively for A homopolymers. For G homopolymers this drop off occurred around 10 bp for both PCR-free and PCR-plus (note that in the case of C homopolymers these thresholds were 12 and 10 bp for PCR-free and PCR-plus respectively, see **Supp Fig 9**).

For INDELs, we also observed an unexpected increase from baseline (i.e., higher precision relative to non-homopolymers) in both A and G homopolymers for short lengths of between 4 and ~10 bp, with a peak around 8 bp. Because the EBM score is a function of both correct and incorrect variant call rates, we hypothesized that the rate of correct calls increases faster than the rate of sequencing errors in short homopolymer regions. Supporting this hypothesis, the ratio of correct to incorrect calls was higher for short homopolymers than for non-homopolymers, which may be caused by the higher rate of true INDEL variants in homopolymers (e.g., 39% of benchmark INDELs are in homopolymers 7 to 10 bp, while only 1.7% of the benchmark regions are in these homopolymer regions, **Supp Table 3**). Indeed, when plotting the correct and incorrect call rates per base pairs covered by each homopolymer size, we saw that the correct call rate increased faster than the incorrect call rate for small homopolymers. As the homopolymer length increased, the incorrect call/bp rate increased more than the correct call/bp rate, which was reflected in the decreased EBM score. Interestingly, both rates decreased for very large homopolymers, likely because large homopolymers are more likely to be excluded from the v4.2.1 GIAB benchmark, reflecting a limitation of the current training dataset (**Supp Fig 10**). These results explain the increase in EBM score for short homopolymers, followed by a decrease, before flattening out due to the small number of very long homopolymers included in the v4.2.1 benchmark. This deep-dive into a counter-intuitive result highlights both the challenges in interpreting the model's results, particularly that it is modeling the ratio of correct to incorrect variant calls rather than the incorrect call rate per genomic bp, as well as its power in identifying unexpected associations of features with error rates.

We also noticed that the EBM INDEL predictions had some sharp downward peaks for particular values of segmental duplication length and identity (**Supp Fig 11a**). When examining variants in the 2 largest peaks near 20 kbp, we found that they were caused by segmental duplication between chr7:142,450,000-142,526,000 on GRCh38 inside the T cell receptor beta locus. This region has a known issue in GRCh38, and the patch contains a ~20 kbp insertion, which is an extra tandem copy of the segmental duplication and causes many incorrect variant calls in Illumina and HiFi (**Supp Fig 11b**). It also intersects with 2 types of problematic reference regions identified in the recent T2T variants work: GRCh38 collapsed duplications and regions with many variants filtered by gnomAD due to abnormal inbreeding coefficient, both of which annotate regions with higher likelihood of falsely calling a variant due to reads from extra copies of the region that are in most genomes but missing from the reference.[5] This result highlights a strength of this model to identify unexpected relationships between features and errors, and also suggests the possibility of adding new features associated with reference errors to future versions of the model.

These results indicate that these models can be used to predict precision with respect to a meaningful, interpretable genomic context, which in turn could be useful in defining more accurate stratifications.

Comparison of EBM performance to DeepVariant performance within candidate regions

We next compared the accuracy of StratoMod's precision prediction to DeepVariant's, for the candidates generated by DeepVariant. We first examined the calibration of DeepVariant's genotype quality score (GQ) (**Supp Fig 12a**) and StratoMod's probability score (**Supp Fig 12b**). As expected from DeepVariant's richer information used for classification, it provided useful phred-based quality scores up to empirical scores of >50 (1 in 100,000 error rate) vs. the v4.2.1 benchmark, though it was somewhat overconfident for INDELs. StratoMod provides well-calibrated scores up to about 35 (1 in 3000 error rate) for SNVs and 25 (1 in 300 error rate) for INDELs. In **Supp Figure 12b**, we assign a label on "1" to any StratoMod probability > .5 and

“0” otherwise. We then made a Venn diagram showing intersections between DeepVariant and StratoMod predicted precision against the benchmark when combining SNVs and INDELs for PCR-free and PCR-plus Illumina. 99.4% of the benchmark variants were classified as correct calls by both StratoMod and DeepVariant. Of the remaining 0.6%, most (36,675) were classified incorrectly as incorrect calls by StratoMod and correctly as correct calls by DeepVariant, whereas 1,765 were correctly classified as correct calls by StratoMod and not DeepVariant. In addition, StratoMod incorrectly classified 44,629 variants as correct that were correctly filtered by DeepVariant, many more than the 3,930 uniquely incorrect correct calls in DeepVariant. When intersecting these uniquely mis-classified variants with features, DeepVariant uniformly performed better, but ~25% of incorrect variants uniquely classified as correct by DeepVariant were in regions difficult to map with 250 bp reads, suggesting DeepVariant may benefit from additional genome mappability features.

Comparison of EBMs to other commonly used models

We compared the performance of an EBM model to XGBoost (XGB), Random Forest (RF), Logistic Regression (LR), and Decision Tree (DT) models in classifying variants as errors using the same training and testing data. In all cases, performance was comparable when examining the area under the curve (AUC) for the receiver operator characteristic (ROC) curve and precision-recall (PR, note that “precision” and “recall” here refer to classification performance and not the predicted precision or recall represented by StratoMod’s output) curves (**Supp Table 4**). Note that the RF SNV model did not complete within the constraints imposed by our compute cluster.

While similar, StratoMod performed slightly better than both DT and LR models and slightly worse than RF and XGB models for both SNVs and INDELs. This is not surprising considering that RF and XGB are much more flexible than EBMs, and EBMs in turn are more flexible than LR models. DT models might be more flexible than EBMs given that they have less restrictions when building trees, but DT models also tend to be brittle as they are not ensemble models (unlike EBMs). Notably, the models that did perform slightly better than EBMs were also blackbox models (e.g. unable to be inspected analogously to EBMs).

These data demonstrated that for this use case, using EBMs for the classification algorithm in StratoMod performed similarly to other commonly used models. Slight performance benefits may be had with blackbox models such as random forest or XGBoost, albeit with a loss of interpretability and potentially much higher compute requirements.

Supplemental Note 3

The data for **Figure 5** was generated using the following command:

```
bcftools isec -p  <out_dir> -O z --threads 8 gnomad.vcf.gz clinvar.vcf.gz
```

The `clinvar.vcf.gz` file was the downloaded ClinVar release vcf modified to have the clinical significance in the INFO column and StratoMod's predicted probability in the SAMPLE/FORMAT fields.

The isec command above produces 4 files corresponding to the overlap between the two vcf files. The last two files (named 0002 and 0003) are Supplemental Files 5 and 6 respectively for INDELs and 7 and 8 for SNVs. Files 0002 and 0003 denote shared variants between gnomAD and ClinVar respectively.

Supplemental Note 4

Mathematical interpretation of StratoMod's predictions

StratoMod's probability may be interpreted in several ways depending on the variant calling results that are used to train the model. These are briefly outlined in **Figure 1c** but are discussed more precisely here.

When comparing a query variant callset to a benchmark callset, a variant can be labeled as a true positive (TP, "in both"), false positive (FP, "only in query"), or false negative (FN, "only in benchmark"). Since StratoMod is a binary classifier, it can only assess two of these outcomes at once, which means either we need to choose two labels, or we need to combine two labels. Logically, TP is always the positive class, and the negative class can either be FN, FP, or both.

Subsetting or combining these labels has different consequences for how one must interpret StratoMod's output. Considering only TP and FN, this means StratoMod is predicting "the likelihood of a variant being in both the query and benchmark given that it is in the benchmark." In mathematical terms, this is `P(Q|G)` where `Q = variant in query` and `G = variant in genome` (where a benchmark is a special genome for which the truth is known and thus a model can be trained). This follows because `G` can only have variant calls labeled as TP or FN, and `Q` can only have TP calls (the query also has FP calls but these are subset out of the training set). Furthermore, this is analogous to recall, since `recall = TP / (FN + TP)`. Thus subsetting StratoMod's training set to TP and FN leads to its output reflecting the predicted recall of a variant.

This interpretation should not change when making predictions using a ClinVar VCF which is not a benchmark itself. In this case, StratoMod is regarding each ClinVar variant as if it exists in the target genome being sequenced via the pipeline that generated the training data for StratoMod, and then predicting how likely it will be detected in the query.

By similar reasoning, subsetting to FP and TP leads to StratoMod's output reflecting predicted precision. This is exactly the reverse of the predicted recall above. Since TP can only be in the benchmark and FP and TP can be in the query, it follows that StratoMod's prediction is "the likelihood of a variant being in the genome given it is in the query" or `P(G|Q)`. Since `precision = TP / (TP + FP)`, StratoMod's output represents predicted precision.

Including FN and FP in the negative class is less intuitive but still has a useful interpretation. In this case, StratoMod is predicting "the likelihood of a variant being in both the benchmark and query given it is in either." Probabilistically this is `P(Q and G|Q or G)` and equivalently `TP / (TP + FP + FN)`, which is the Jaccard index (the "overlap" between sets).

It should be noted that the probabilities above are conditional and not the same as the probability of seeing a given variant calling result. For example, the "probability of a true positive" (in both benchmark and query) is `P(Q and G)`, which StratoMod cannot produce. However, it is easy to derive this from StratoMod's predicted recall if one has a prior likelihood of the variant existing in the genome, `P(G)`. In that case, `P(Q and G) = P(Q|G) * P(G)` by the chain rule where `P(Q|G)` is StratoMod's output. One could similarly derive the probability of a false negative by `P(!Q and G) = P(!Q|G) * P(G) = (1 - P(Q|G)) * P(G)`.

Supplemental figures:

A.

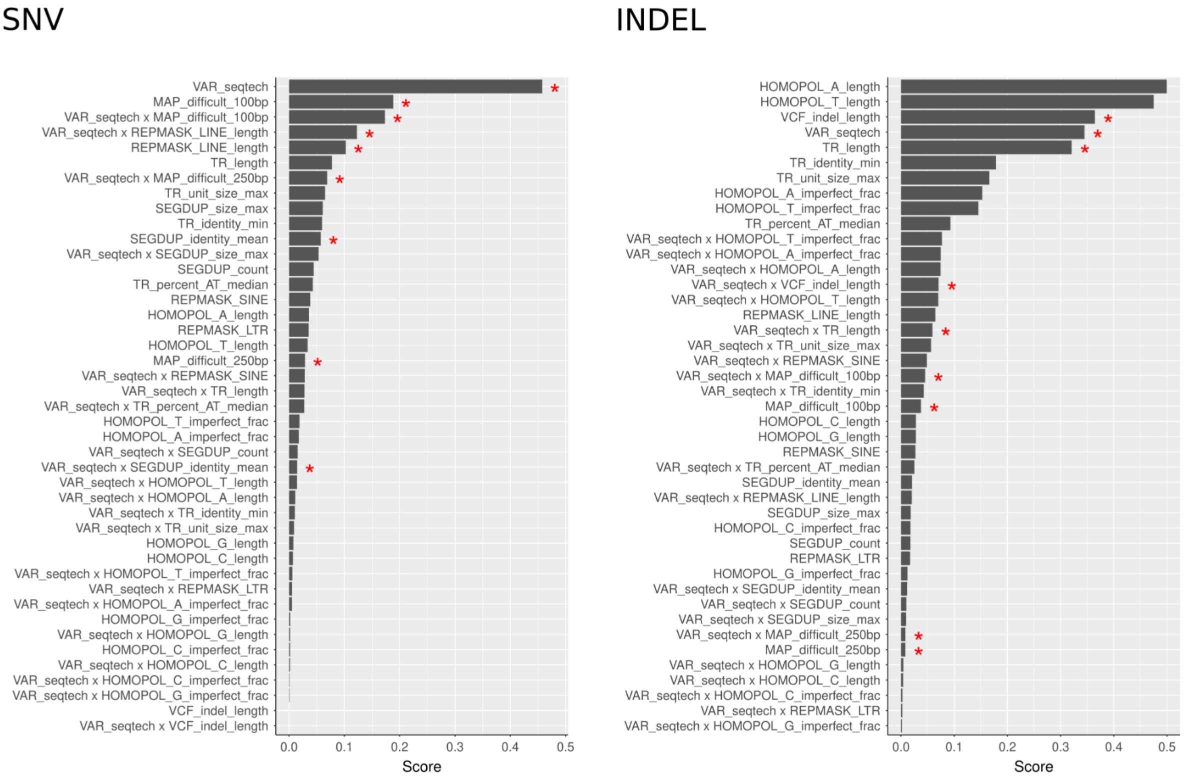

B.

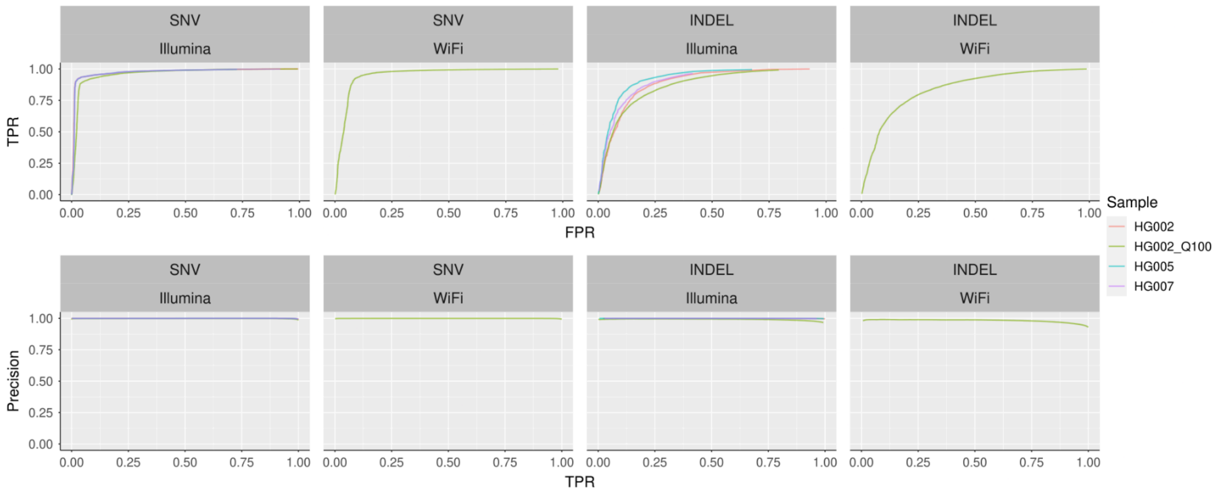

Supplemental Figure 1: Global importance plots (a) and performance curves (b) for Hifi vs Illumina Clinvar experiment. TPR = true positive rate. Train was done on 80% of "HG002_Q100" and test was done on 20% of "HG002_Q100" and the whole of the other genomes, which were all in terms of the v4.2.1 Genome in a bottle benchmark. Note: "VAR_seqtech" is a different name for "VCF_input" as described in **Supplemental Table 1**. Note that "precision" and "recall" (aka true positive rate, TPR) here are classification performance metrics and are not the same as predicted precision or recall represented by StratoMod's output.

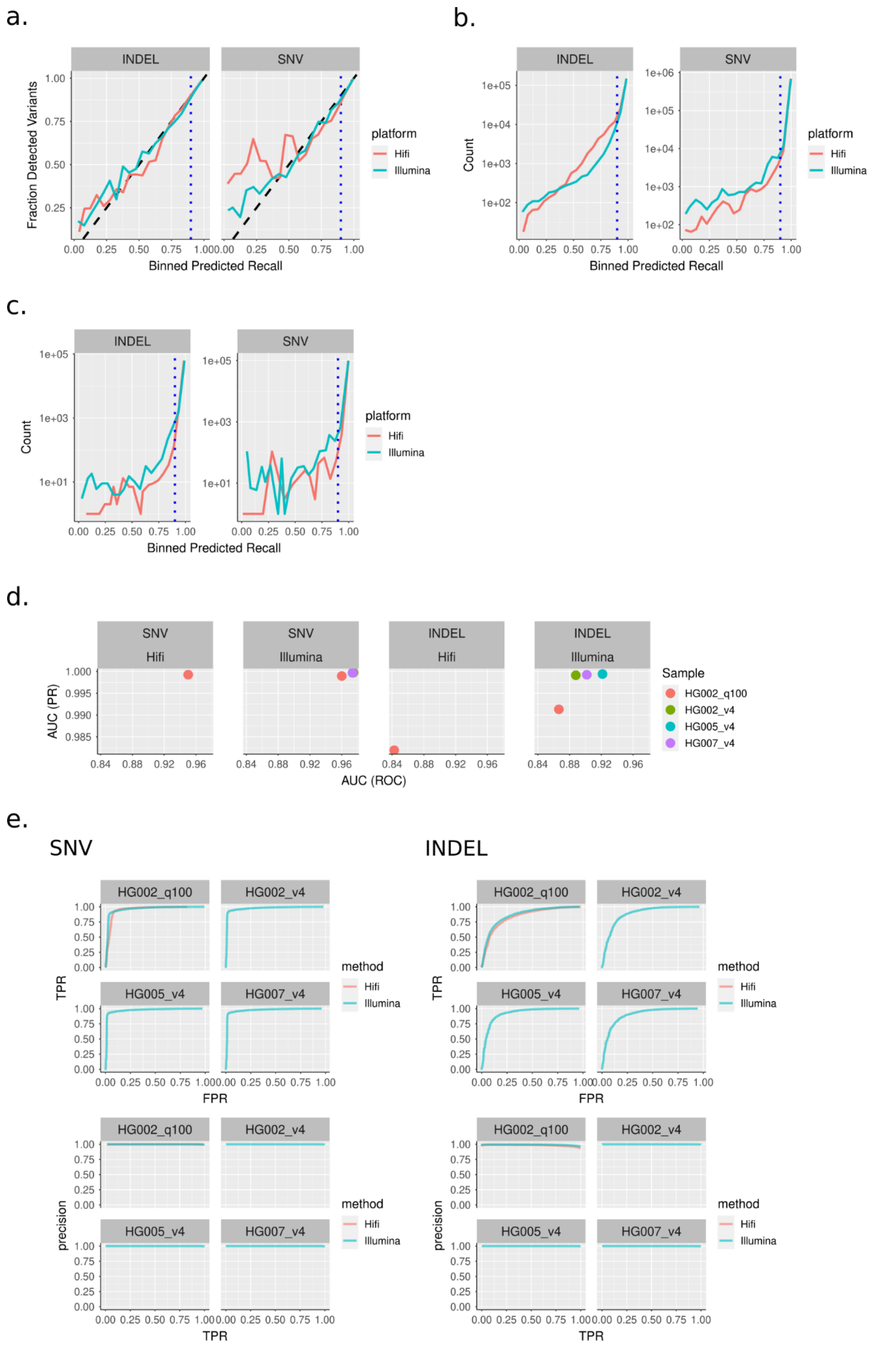

Supplemental Figure 2: Performance metrics for Hifi vs Illumina recall model. A) calibration curves where the dotted black line is perfect calibration and blue dotted line is the 90% threshold used in the analysis. B-C) Counts for each bin shown in (A) for test data set (B) and clinvar variants (C) D) AUC under precision-recall (PR) and receiver-operator (ROC) curves for all models. D) Raw ROC and PR curves for each genome and platform. Note that "precision" and "recall" (aka true positive rate, TPR) here are classification performance metrics and are not the same as predicted precision or recall represented by StratoMod's output.

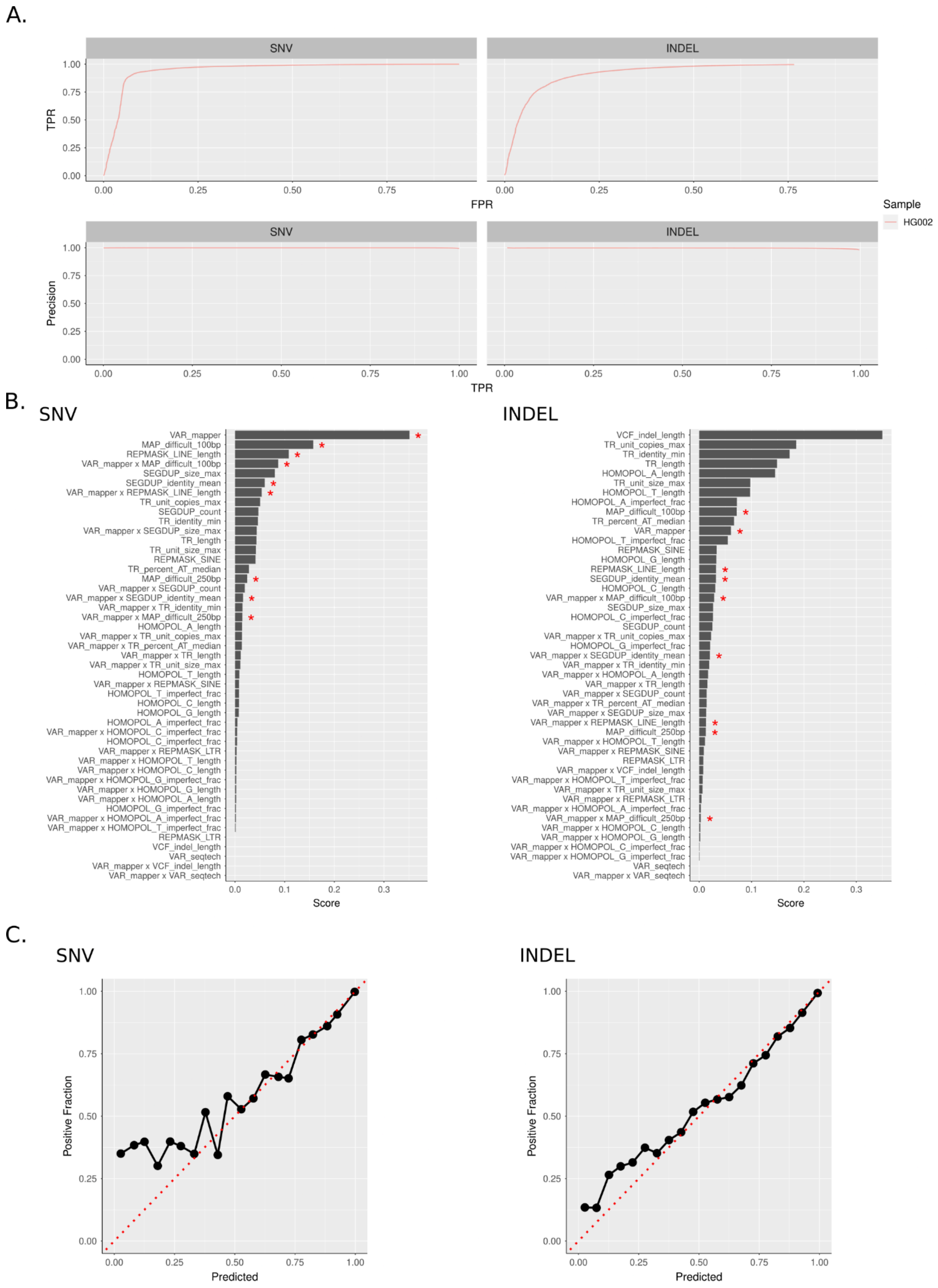

Supplemental Figure 3: Performance metrics for Element VG vs BWA recall model a) ROC and PR curves b) feature importance plots c) calibration curves (dotted line is perfect calibration). Note: “VAR_mapper” is a different name for “VCF_input” as described in **Supplemental Table 1**.

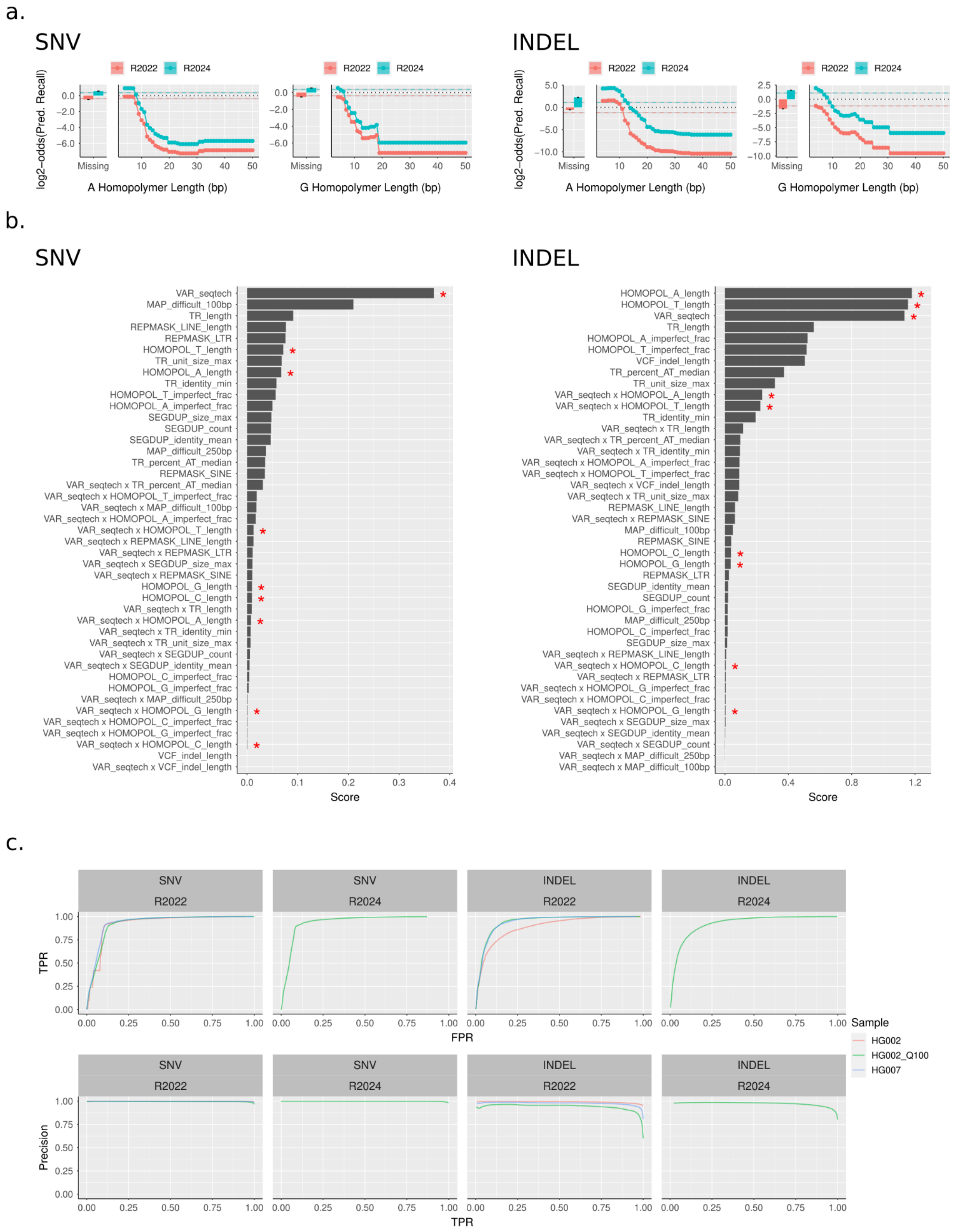

Supplemental Figure 4: StratoMod utilized to compare different versions of emerging technologies (Ultima versions 2022 vs 2024) in terms of their predicted recall. a) homopolymer profiles for INDELs and SNVs b/t old and new. Error bars and ribbons around step plots are model error. b) global feature plots for models and c) performance curves for each model. Note: “VAR_seqtech” is a different name for “VCF_input” as described in **Supplemental Table 1**. Note that “precision” and “recall” (aka true positive rate, TPR) here are classification performance metrics and are not the same as predicted precision or recall represented by StratoMod’s output.

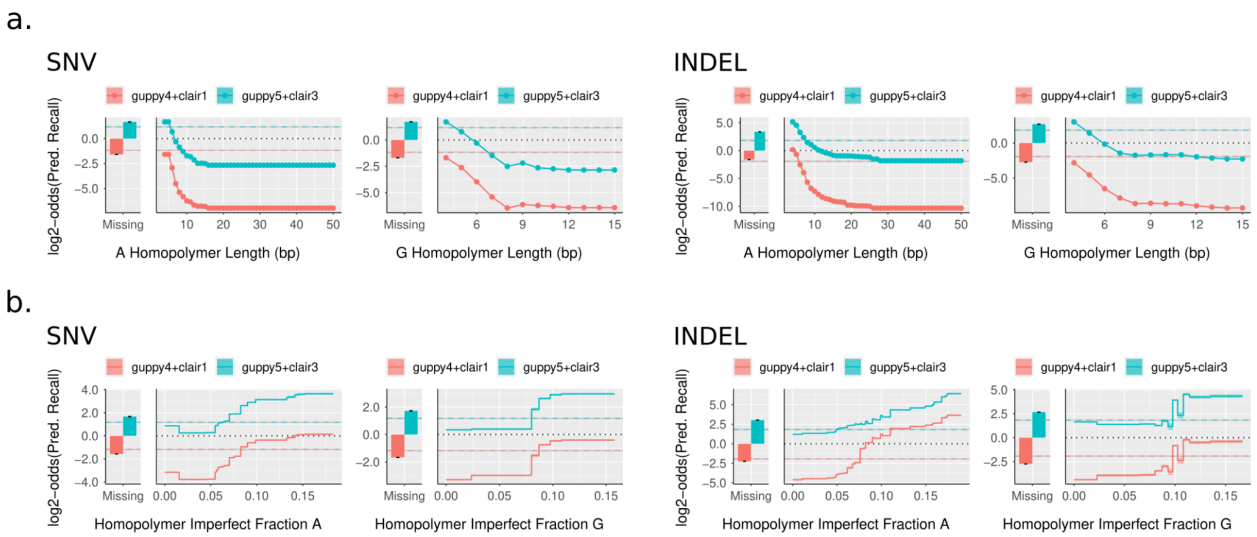

Supplemental Figure 5: StratoMod utilized to compare different versions of ONT base/variant callers (guppy4+clair1 and guppy5+clair3) in terms of their predicted recall for SNVs and INDELs using HG003 as the benchmark, specifically for a) homopolymer length profiles and b) homopolymer imperfect fraction profiles. Error bars and ribbons around step plots are model error

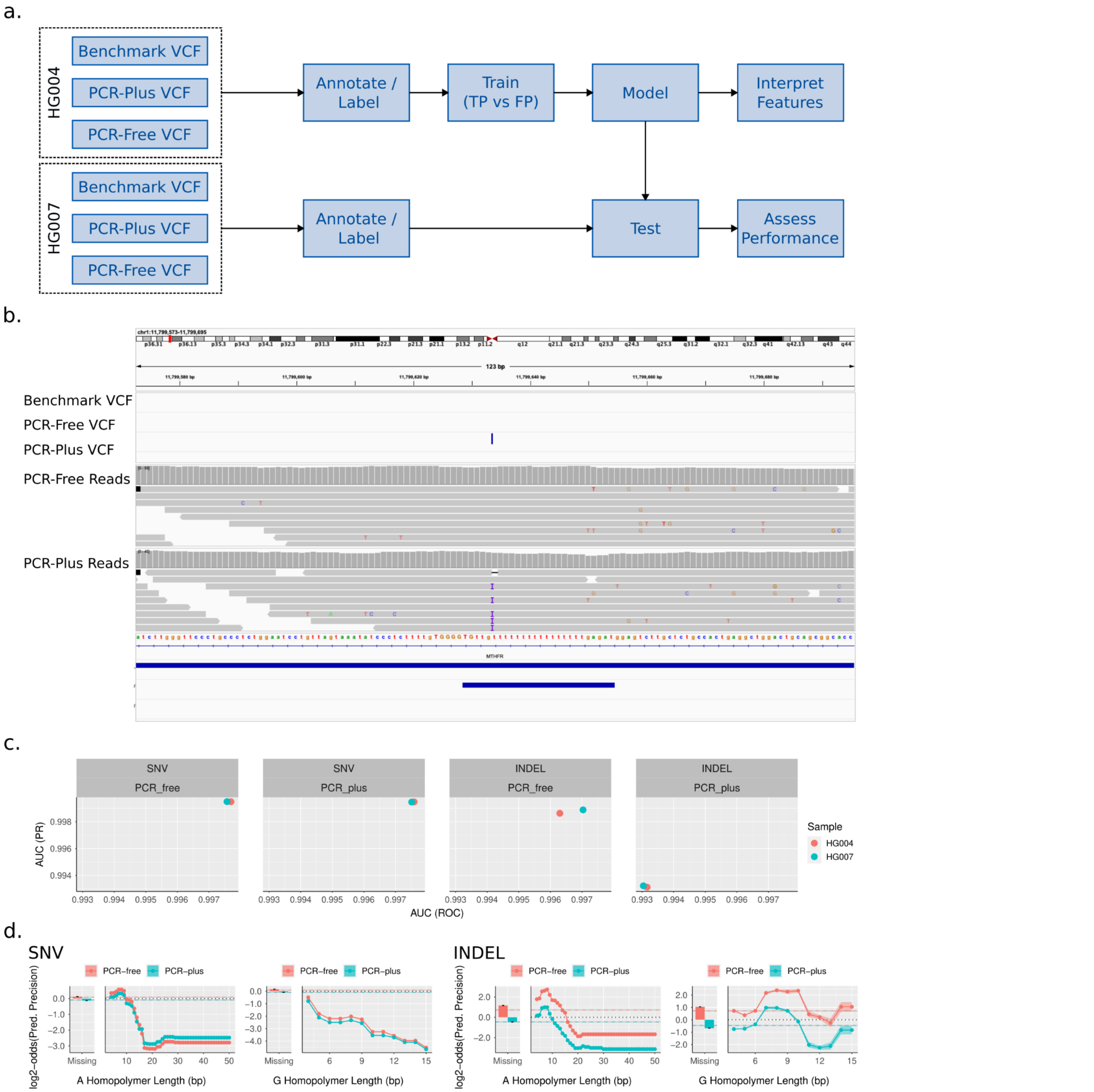

Supplemental Figure 6: StratoMod revealed context-specific regions where incorrectly called variants are likely to occur and show relative performance of PCR-free and PCR-plus technologies. a) Overview of experimental setup. Two VCFs from PCR-free and PCR-plus were compared to the GIAB benchmark, concatenated, and annotated before fitting SNVs and INDELs in the EBM framework. HG005 was annotated and used to test the EBM model. b) IGV session depicting an incorrect variant call identified by this model c) performance characteristics of HG004 (train) and HG007 (test). d) EBM plots showing A and G homopolymer profiles. The x axis in all plots was truncated to only show homopolymers <50bp and <15bp for A and G respectively. The bar plots on the left of each plot show the value for non-homopolymers (“missing”). The y axis in the top row is the log odds of a predicted correct variant call. Each colored dotted line in the top row is the “baseline” error rate for the corresponding sequencing technology. Error bars and ribbons around step plots are model error

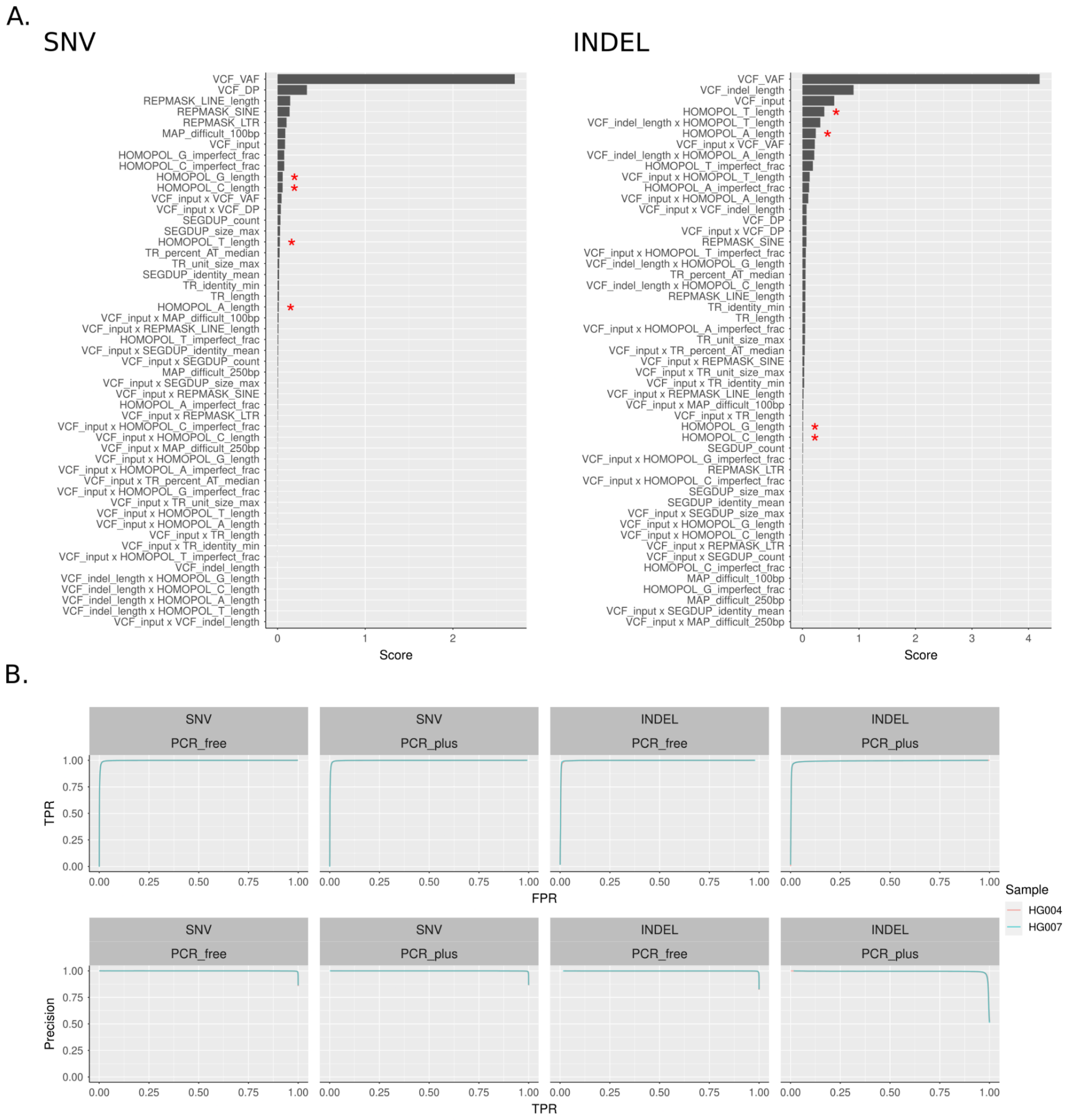

Supplemental Figure 7: Performance of PCR-free/plus models. a) global feature plots and b) ROC and PR curves. Note that “precision” and “recall” (aka true positive rate, TPR) here are classification performance metrics and are not the same as predicted precision or recall represented by StratoMod’s output.

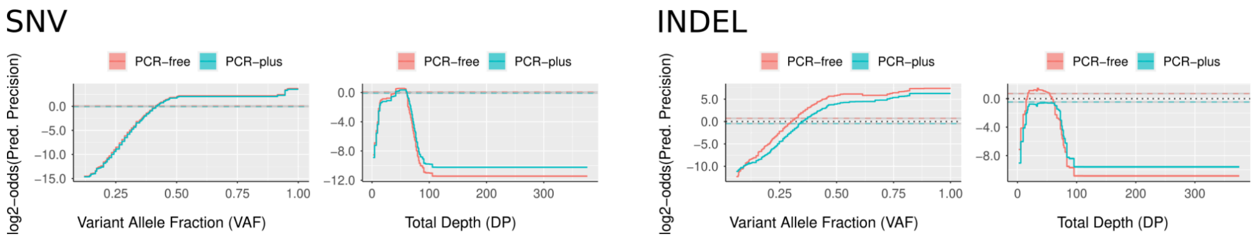

Supplemental Figure 8: VAF and DP feature plots for both SNV and INDEL precision models.

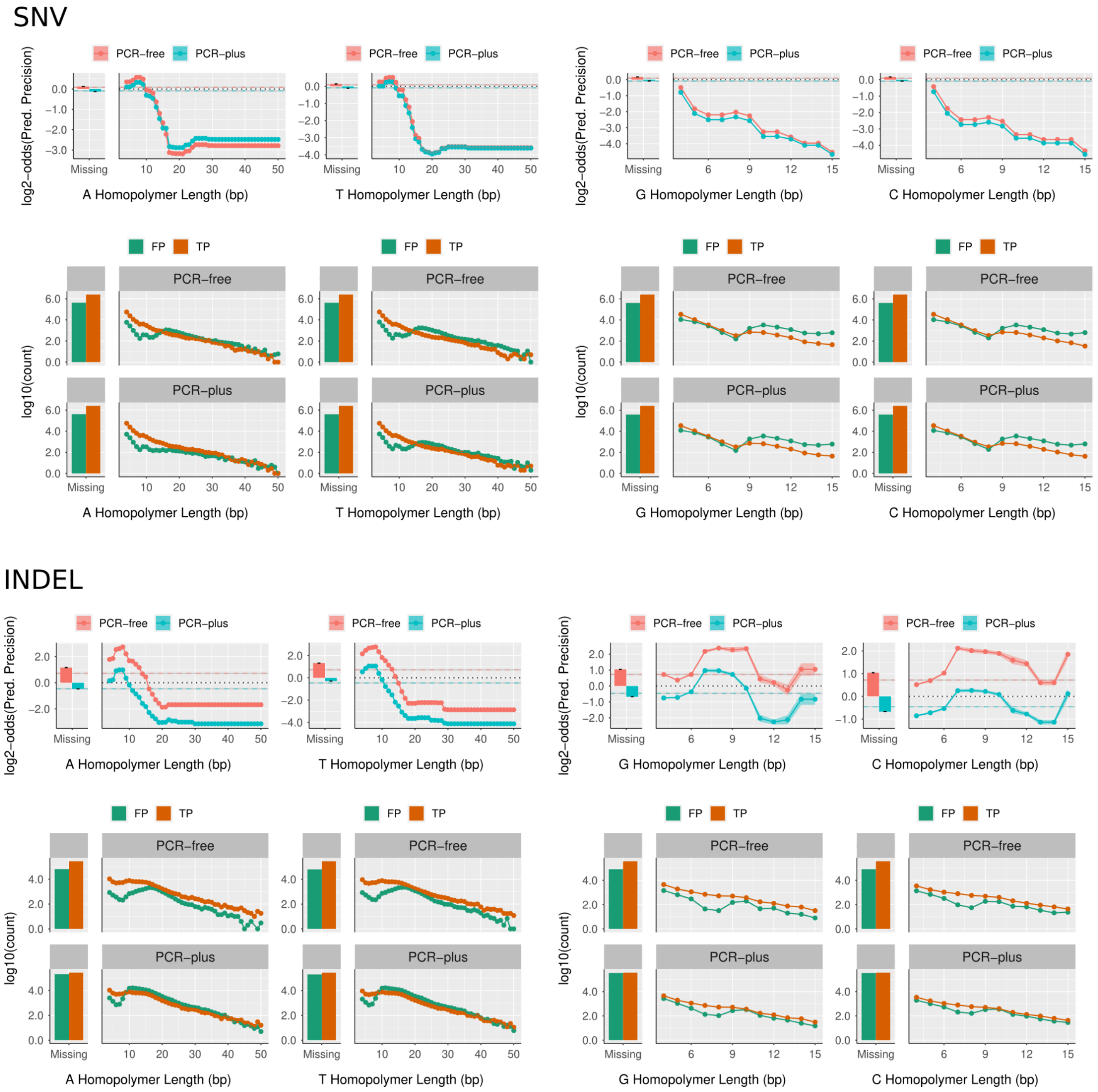

Supplemental Figure 9: Complementary homopolymers for the precision model for both SNVs and INDELs.

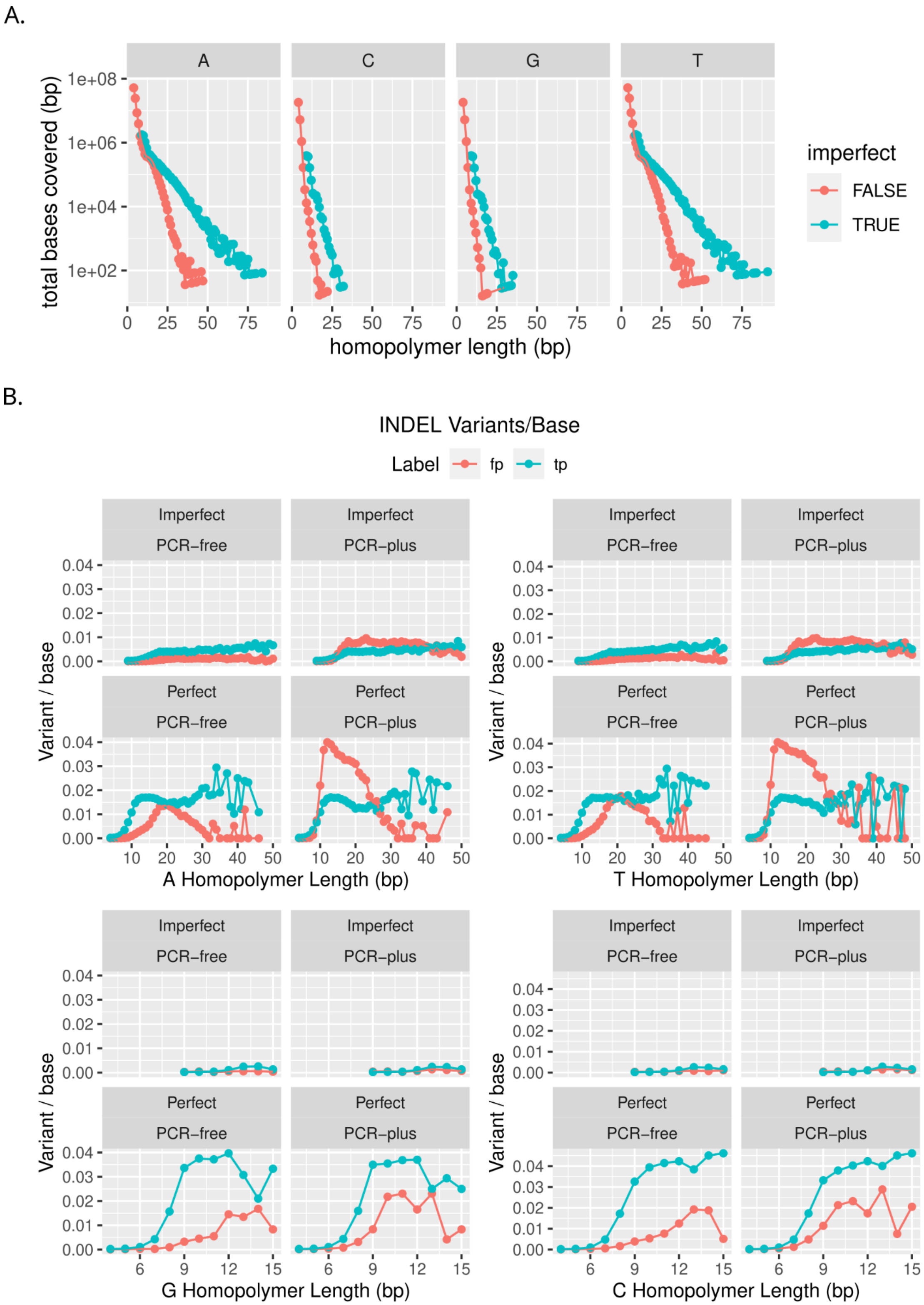

Supplemental Figure 10: Decomposition of homopolymer features for INDELs to better understand the EBM scores. A) total bases covered for each base homopolymer stratified by length B) number of variants normalized to total bases covered (from A) for each base stratified by length

a.

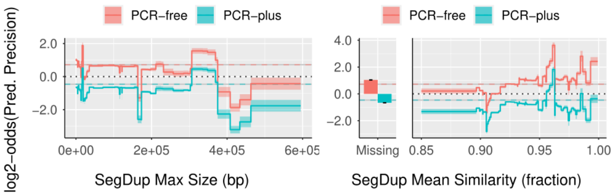

b.

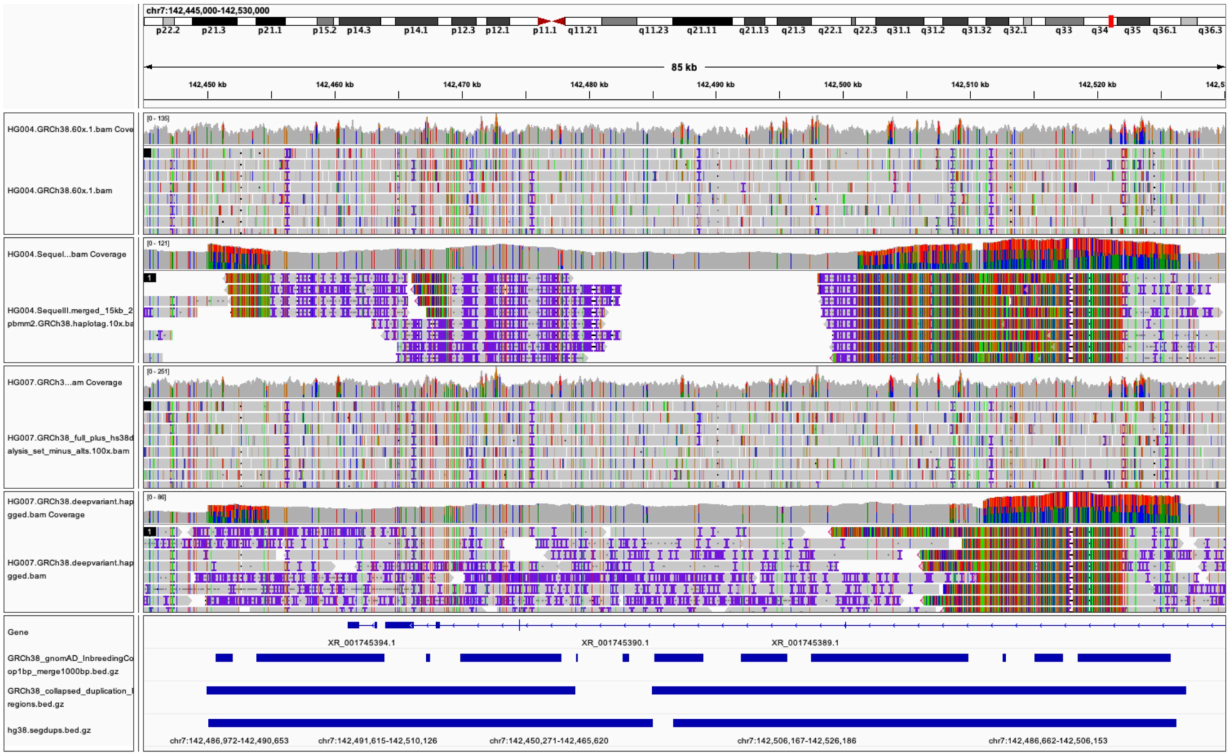

Supplemental Figure 11: Explainable segdup features facilitated identification of mapping errors in known hard regions. A) The INDEL feature profiles for the precision model for segdup identity and length (note the peak at ~20k). B) IGV screenshot showing incorrectly called variants due to mapping errors from a duplicated region in HG004/HG007 due to an error in GRCh38 missing a copy of genes in this T cell receptor beta locus.

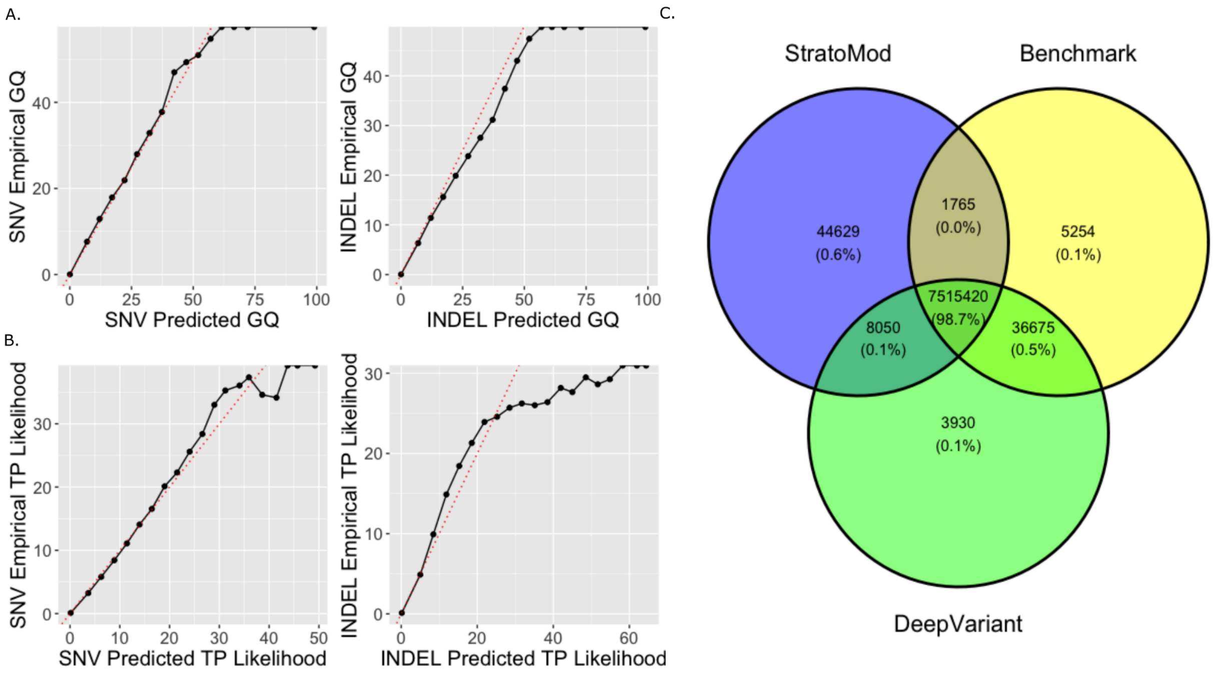

Supplemental Figure 12: Comparison of Stratomod calibration and accuracy relative to deep learning-based method DeepVariant. a) Predicted genotype quality score (GQ) from DeepVariant plotted against an empirically derived GQ measure, -10*log10(FP/(FP+TP)), using the GIAB v4.2.1 small variant benchmark. b) PHRED-scaled plots depicting calibration for how well StratoMod confidently predicts correct variant calls (or values near 1 for its predictions) similar to a typical genotype quality score c) Comparison of StratoMod and DeepVariant performance against the benchmark values for the candidate sites from DeepVariant used in our model.

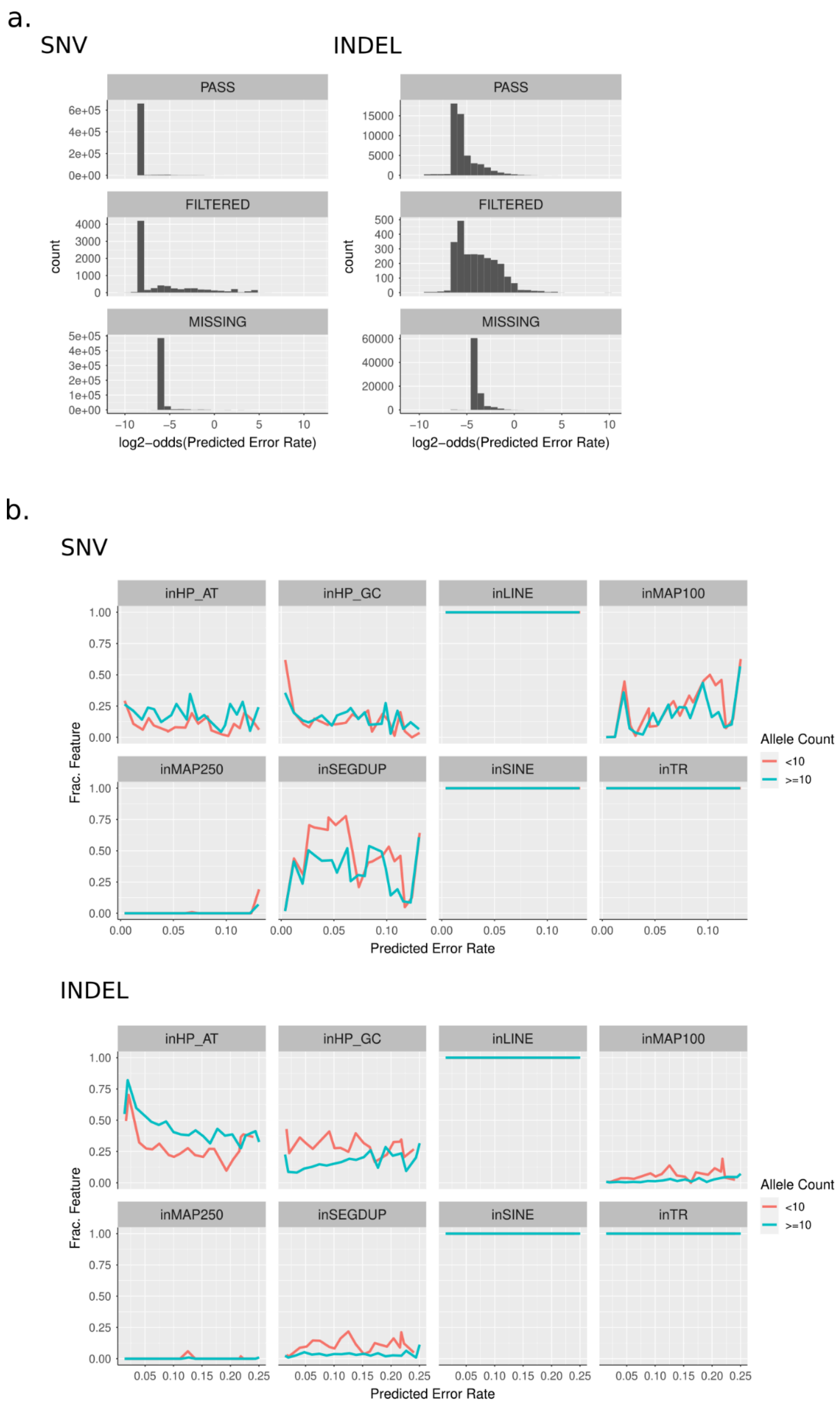

Supplemental Figure 13: a) Histograms of Clinvar variants and their predicted error rate that either corresponded to a PASS gnomAD variant (“PASS”), filtered gnomAD variant (“FILTERED”) or no gnomAD variant (“MISSING”). b) Variants binned by predicted error rate and stratified by region type and allele count. Y axis is the fraction of variants per bin that are within the denoted region type.

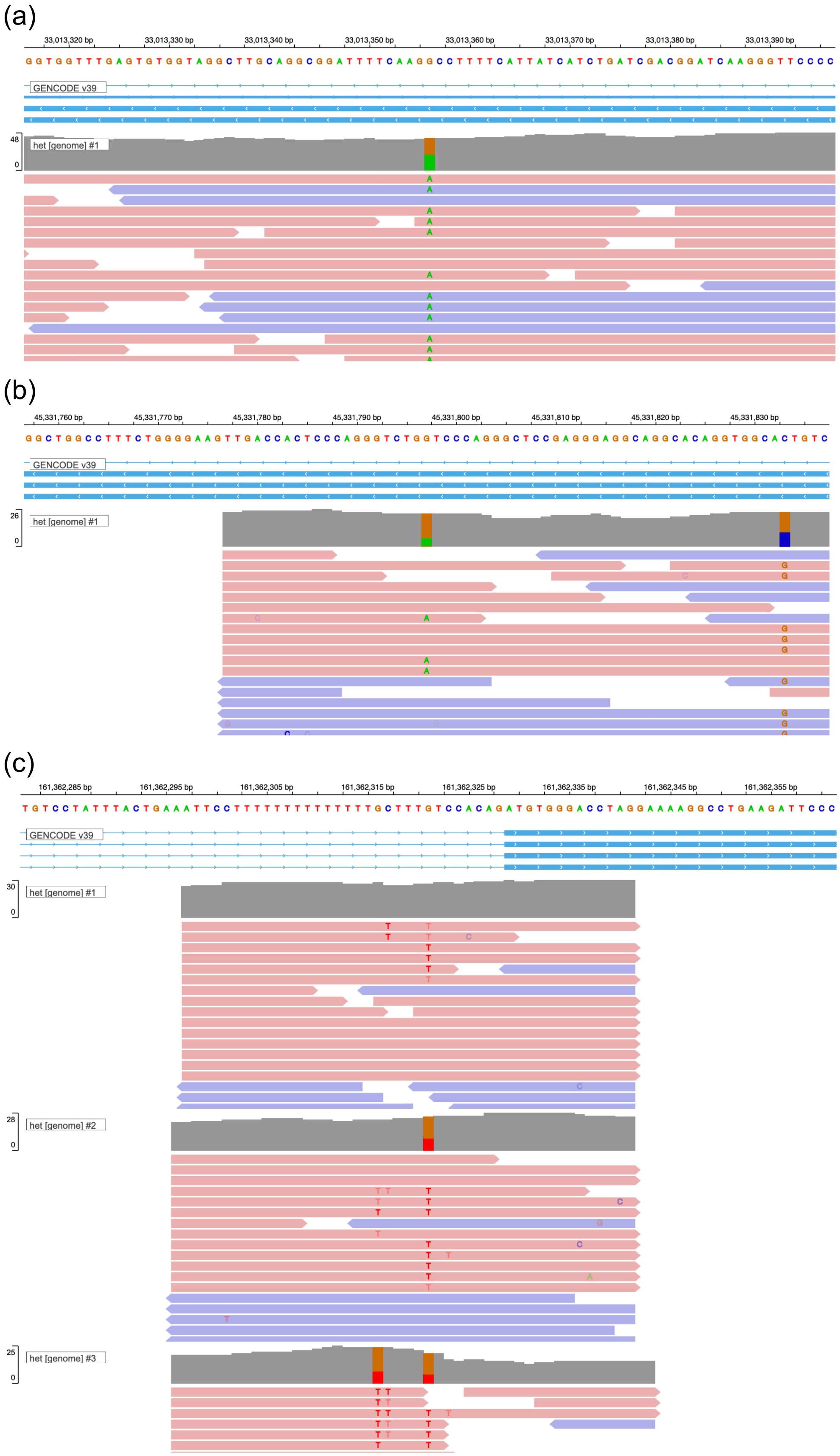
(a)
33,013,320 bp
33,013,330 bp
33,013,340 bp
33,013,350 bp
33,013,360 bp
33,013,370 bp
33,013,380 bp
33,013,390 bp
GENCODE v39
48
0
het [genome] #1
(b)
45,331,760 bp
45,331,770 bp
45,331,780 bp
45,331,790 bp
45,331,800 bp
45,331,810 bp
45,331,820 bp
45,331,830 bp
GENCODE v39
26
0
het [genome] #1
(c)
161,362,285 bp
161,362,295 bp
161,362,305 bp
161,362,315 bp
161,362,325 bp
161,362,335 bp
161,362,345 bp
161,362,355 bp
GENCODE v39
30
0
het [genome] #1
28
0
het [genome] #2
25
0
het [genome] #3

(d)

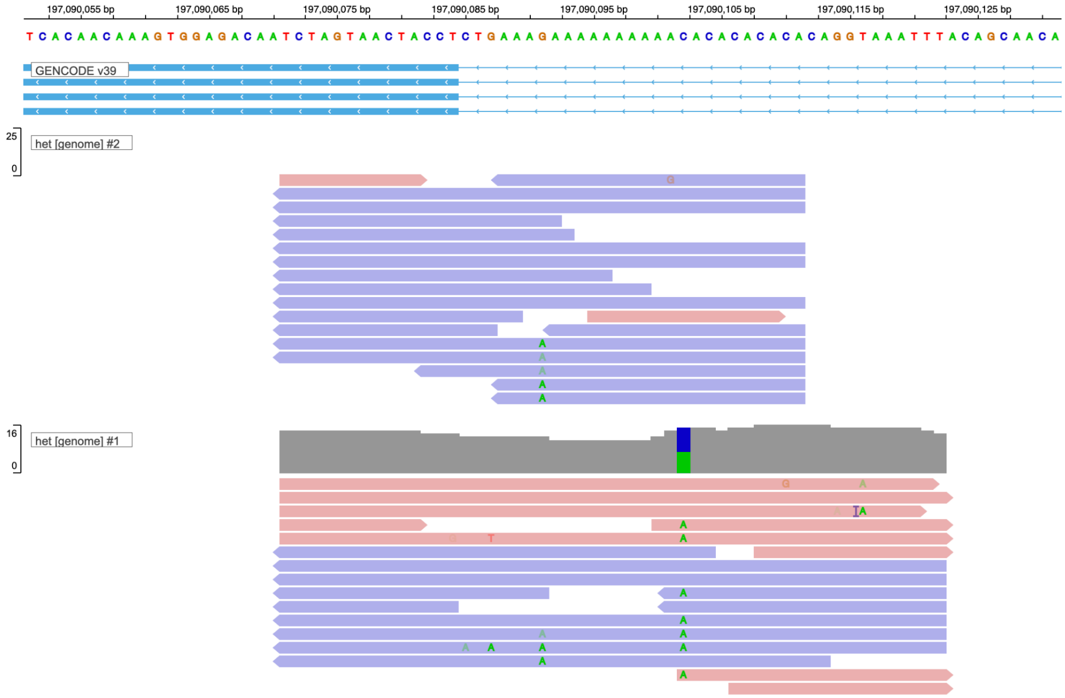

Supplemental Figure 14: Examples of gnomAD variants on GRCh38 chr1 that were filtered despite having a low error rate predicted by Stratomod (score>0.99). Upon curation, these generally fell in two categories: (a-b) variants in a very small number of samples, typically one or two, that appear likely to be true and were likely incorrectly filtered by gnomAD. (c-d) variants with evidence of systematic errors indicated by strand bias in homopolymers, and were generally called in more samples than the first category but still generally <100.

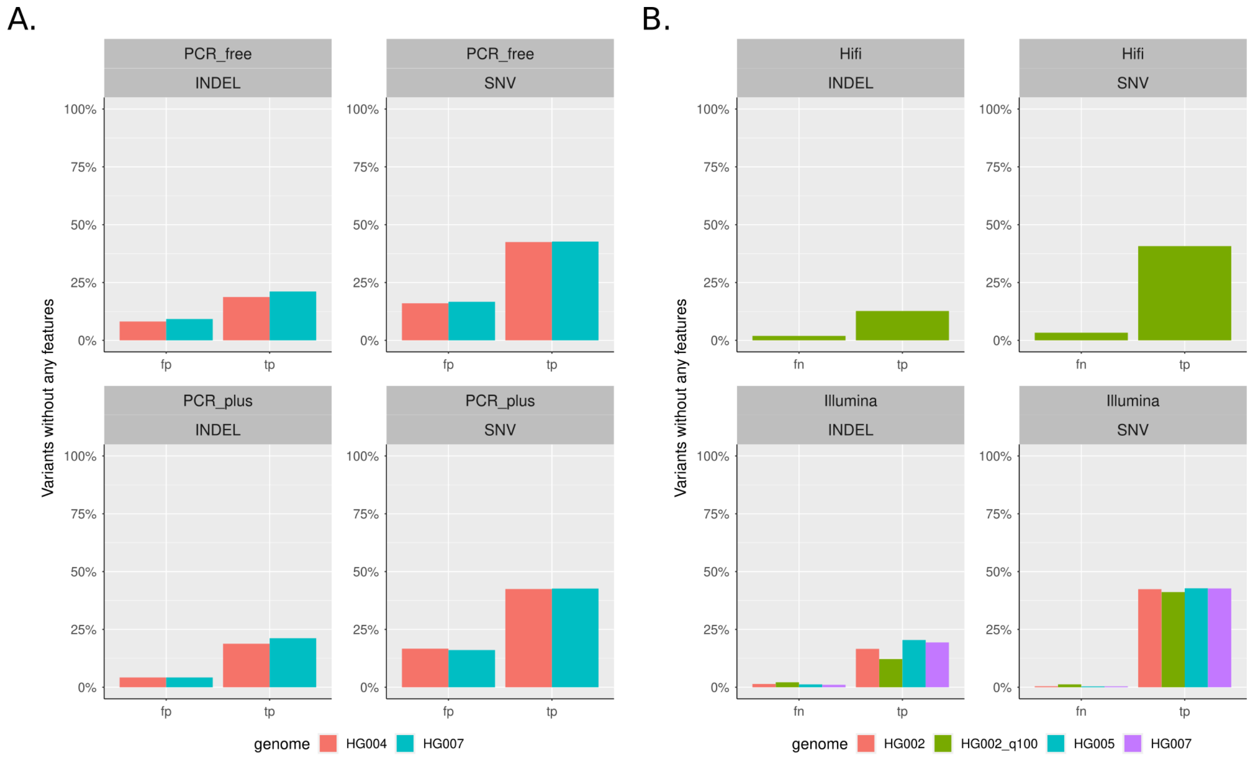

Supplemental Figure 15: Percent of variants which have no coverage by our engineered feature set in the a) Illumina PCR-free/plus precision model and b) the HiFi/Illumina recall model. Note that DP and VAF were excluded from this analysis, as they are expected to cover all variants in the callset but not variants in the benchmark which were missed.

Supplemental Tables

Supplemental Table 1: Overview of features used in each model. Feature names containing a string bracketed like <X> (in red) denote shorthand to describe features that are the same in all but one way.

Feature Name	Description	Domain	Models
VCF_input *	The VCF file corresponding to the origin of the variant; used to represent the different technologies/pipelines, mappers, library preps, etc (eg PCR-free vs PCR-plus or Hifi vs Illumina)	categorical	all
VCF_VAF	The VAF (Variant Allele Fraction) value as recorded in the VCF file by the variant caller	[0-1]	PCR-free /plus only
VCF_DP	The DP (Depth of Coverage) value as recorded in the VCF file by the variant caller	[1-inf]; integer	PCR-free /plus only
VCF_indel_length	The length of an INDEL (0 in the case of SNVs)	[-inf-inf]; integer	all
HOMOPOL_<base>_length (where <base> is either A, T, G, or C)	The length of a homopolymer region	[1-inf]; integer	all
HOMOPOL_<base>_imperfect_frac (where <base> is either A, T, G, or C)	The fraction of bases in a homopolymer that are not <base>	[0-1]	all
TR_length	The length of a tandem repeat region	[1-inf]; integer	all
TR_unit_size_max	The size of a single repeat unit that is repeated TR_unit_copies times in a tandem repeat region. For overlapping regions the maximum was used.	[1-inf]; integer	all
TR_identity_min	The similarity of each unit across the tandem repeat (corresponds to 'perMatch' in the TRF-based UCSC simple repeats database). For overlapping regions the minimum was used.	[1-inf];	all
TR_percent_AT_median	The percentage of the tandem repeat region which is an A or T base	[0-100]; integer	all
REPMASK_SINE	TRUE is the variant intersects with a SINE	boolean	all
REPMASK_LTR	TRUE if the variant intersects with an LTR	boolean	all

REPMASK_LINE_length	The length of the LINE in which this variant is found	[1-inf]; integer	all
MAP_difficult_<X>bp (where X is either 100 or 250)	TRUE if the variant intersects with a hard-to-map region for read pairs of length X.	boolean	all
SEGDUP_size_max	The length of the segmental duplication region. For overlapping regions, the maximum was used.	[1000-inf]; integer	all
SEGDUP_identity_mean	The similarity of this segmental duplication to others (corresponds to “fracMatchIndel” from the genomic super dups database). For overlapping regions the mean was used.	[0-1]	all
SEGDUP_count	The number of segmental duplications overlapping this region.	[1-inf]; integer	all

* The feature “VCF_input” was renamed in later versions of StratoMod to make it less abstract. In some models this feature is named “VAR_seqtech” or “VAR_mapper.” Each of these has the same meaning; they denote that the VCF files used for training differ in the process used to generate them, and this variable is meant to capture this difference. For the sake of brevity we are only referring to these by the name “VCF_input” in this table and the main text. The few figures where this name change applies are also footnoted for clarification.

Supplemental Table 2: Model training summary

N Rows	Percent Positive Class	Variant Type	Subsets	Prediction
2939508	94.4	INDEL	Hifi = 1452626; Illumina = 1486882	recall
11412592	98.83	SNV	Hifi = 5718674; Illumina = 5693918	recall
2406562	97.62	INDEL	VG = 1202820; BWA = 1203742	recall
11407256	99.24	SNV	VG = 5704070; BWA = 5703186	recall
2640324	70.16	INDEL	R2024 = 1366776; R2022 = 1273548	recall
11388272	97.11	SNV	R2024 = 5689340; R2022 = 5698932	recall
2375368 *	63.69	INDEL	PCR-free = 929278; PCR-plus = 1446090	precision
12311190 *	85.95	SNV	PCR-free = 6177338; PCR-plus = 6133852	precision
383191	92.45	INDEL	NA	Jaccard index
1429350	98.06	SNV	NA	Jaccard index

* All used the GIAB assembly based small variant benchmark from the T2T-HG002-Q100v0.9 assembly aligned to GRCh38 under https://ftp-trace.ncbi.nlm.nih.gov/ReferenceSamples/giab/data/AshkenazimTrio/analysis/NIST_HG002_DraftBenchmark_defrabbV0.011-20230725/ except for these, which used the GIAB v4.2.1 small variant benchmark from https://ftp-trace.ncbi.nlm.nih.gov/ReferenceSamples/giab/release/AshkenazimTrio/HG002_NA24385_son/NISTv4.2.1/GRCh38/

Supplemental Table 3: Homopolymer TP likelihood increase for length 7-10bp

**Homopolymer length**	**Number of 1 bp INDELs**	**Number of bp covered by homopolymer + 5 bp slop on each side**
4 to 6 bp	66134	747,661,356
7 to 11 bp	80261	47,824,121
>11 bp	31624	17,659,926
All benchmark regions	204853	2,765,733,593

Supplemental Table 4: Comparison of EBM performance to those of other popular machine-learning methods. OOM: out of memory

**Variant**	**Metric**	**StratoMod**	**DT**	**LR**	**RF**	**XGB**
INDEL	ROC	99.5%	99.2%	99.2%	99.8%	99.8%
INDEL	PR	99.7%	99.4%	99.5%	99.9%	99.9%
SNV	ROC	99.8%	99.4%	99.5%	OOM	99.9%
SNV	PR	99.9%	99.8%	99.9%	OOM	100.0%

Supplemental Table 5: Software packages and versions

**Tool/package name**	**Version**
rtg-tools (vcfeval)	3.12.1
bedtools	2.30.0
Interpretml (the EBM python package)	0.2.7
samtools	1.14
bcftools	1.19

Supplemental Table 6: VCF files used throughout analysis (all with GRCh38* as reference)

Description	Sample	Coverage (X)	Source
Illumina PCR-Free	HG002	40	https://storage.googleapis.com/brain-genomics-public/research/sequencing/grch38/vcf/hiseqx/wgs_pcr_free/40x/HG002.hiseqx.pcr-free.40x.deepvariant-v1.0.grch38.vcf.gz
Illumina PCR-Free	HG004	40	https://storage.googleapis.com/brain-genomics-public/research/sequencing/grch38/vcf/hiseqx/wgs_pcr_free/40x/HG004.hiseqx.pcr-free.40x.deepvariant-v1.0.grch38.vcf.gz
Illumina PCR-Plus	HG004	40	https://storage.googleapis.com/brain-genomics-public/research/sequencing/grch38/vcf/hiseqx/wgs_pcr_plus/40x/HG004.hiseqx.pcr-plus.40x.deepvariant-v1.0.grch38.vcf.gz
Illumina PCR-Free	HG005	40	https://storage.googleapis.com/brain-genomics-public/research/sequencing/grch38/vcf/hiseqx/wgs_pcr_free/40x/HG005.hiseqx.pcr-free.40x.deepvariant-v1.0.grch38.vcf.gz
Illumina PCR-Free	HG007	40	https://storage.googleapis.com/brain-genomics-public/rese

			arch/sequencing/grch38/vcf/hiseqx/wgs_pcr_free/40x/HG007.hiseqx.pcr-free.40x.deepvariant-v1.0.grch38.vcf.gz
Illumina PCR-Plus	HG007	40	https://storage.googleapis.com/brain-genomics-public/research/sequencing/grch38/vcf/hiseqx/wgs_pcr_plus/40x/HG007.hiseqx.pcr-plus.40x.deepvariant-v1.0.grch38.vcf.gz
PacBio HiFi	HG002	37	https://ftp-trace.ncbi.nlm.nih.gov/giab/ftp/data/AshkenazimTrio/analysis/PacBio_CCS_15kb_20kb_chemistry2_10312019/GRCh38/deepvariant_HG002_GRCh38_15kb_37X_SequelII.vcf.gz
PacBio HiFi	HG005	47	https://ftp-trace.ncbi.nlm.nih.gov/ReferenceSamples/giab/release/ChineseTrio/HG005_NA24631_son/NISTv4.2.1/GRCh38/SupplementaryFiles/inputvcfsandbeds/HG005_GRCh38_1_22_PacBio_HiFi_DeepVariant.vcf.gz
PacBio HiFi	HG007	21	https://storage.googleapis.com/brain-genomics-public/research/sequencing/grch38/vcf/pacbio_hifi

			/HG007.pacbio-hifi.21x.deepvariant-v1.0.grch38.vcf.gz
ClinVar	n/a	n/a	https://ftp.ncbi.nlm.nih.gov/pub/clinvar/vcf_GRCh38/archive_2.0/2022/clinvar_20220812.vcf.gz
Ultima R2024	HG002	40	https://giab-data.s3.amazonaws.com/ultima-GIAB-Feb-2024/DeepVariant_vcfs/NA24385-Z0027.annotated.AF.vcf.gz
Ultima R2022	HG002	40	https://s3.amazonaws.com/ultima-selected-1k-genomes-vcf-only/DeepVariant_vcfs/HG002_005401-UGAv3-1-CACATCCTGCATGTGAT.vcf.gz
Ultima R2022	HG007	40	https://s3.amazonaws.com/ultima-selected-1k-genomes-vcf-only/DeepVariant_vcfs/HG007_004731-UGAv3-33-CATGCAGCGCTAATGA.vcf.gz
ONT guppy4+clair1 **	HG003	40	http://www.bio8.cs.hku.hk/clair3/analysis_result/ont_guppy4/2_coverage_subsampling/clair/hg003_40x_clair_filter_q748.vcf.gz
ONT guppy5+clair3 **	HG003	40	http://www.bio8.cs.hku.hk/clair3/analysis_result/ont_guppy5/2_coverage_subs

			ampling/clair3/hg003_40x_clair3.vcf.gz

* GRCh38 from here except where noted:
https://s3.amazonaws.com/rtg-datasets/references/GRCh38.sdf.zip

** GRCh38 from here which has ambiguous bases to work with these specific VCFs:
http://www.bio8.cs.hku.hk/clair3/analysis_result/ont_guppy5/2_coverage_subsampling/clair3/hg003_40x_clair3.vcf.gz

Supplemental references

1. Gymrek, M. PCR-free library preparation greatly reduces stutter noise at short tandem repeats. *bioRxiv* 043448 (2016) doi:10.1101/043448.
2. Fang, H. *et al.* Reducing INDEL calling errors in whole genome and exome sequencing data. *Genome Med.* **6**, 89 (2014).
3. Dohm, J. C., Lottaz, C., Borodina, T. & Himmelbauer, H. Substantial biases in ultra-short read data sets from high-throughput DNA sequencing. *Nucleic Acids Res.* **36**, e105 (2008).
4. Weissensteiner, M. H. *et al.* Distinct sequencing success at non-B-DNA motifs. *bioRxiv* 2022.06.13.495922 (2022) doi:10.1101/2022.06.13.495922.
5. Aganezov, S. *et al.* A complete reference genome improves analysis of human genetic variation. *Science* **376**, eabl3533 (2022).
