# Supplementary material for: StratoMod: predicting sequencing and variant calling errors with interpretable machine learning
Source: Commun Biol. 2024 Oct 13;7:1316. doi: 10.1038/s42003-024-06981-1 (PMC11471861; doi:10.1038/s42003-024-06981-1)
Supplement: Supplementary file 3 — Description of Additional Supplementary Files [file 42003_2024_6981_MOESM3_ESM.pdf]

Description of Additional Supplementary Files

File name: Supplementary Data 1
Description: list of genes with counts of variants >90% likelihood of being missed as calculated by StratoMod

File name: Supplementary Data 2
Description: list of pathogenic variants with >90% likelihood of being missed by Hifi or Illumina as calculated by Stratomod (used to generate Supplemental File 1)

File name: Supplementary Data 3
Description: Raw INDEL data corresponding to Figure 4c (which only shows SNVs)

File name: Supplementary Data 4
Description: Raw SNV data for Figure 4c

File name: Supplementary Data 5
Description: Raw INDEL data for Figure 5 containing all gnomAD variants that are also in ClinVar

File name: Supplementary Data 6
Description: Raw INDEL data for Figure 5 containing all ClinVar variants that are also in gnomAD

File name: Supplementary Data 7
Description: Raw SNV data for Figure 5 containing all gnomAD variants that are also in ClinVar

File name: Supplementary Data 8
Description: Raw SNV data for Figure 5 containing all ClinVar variants that are also in gnomAD
